# CNN-Based Self-Attention Weight Extraction for Fall Event Prediction Using Balance Test Score

**DOI:** 10.3390/s23229194

**Published:** 2023-11-15

**Authors:** Youness El Marhraoui, Stéphane Bouilland, Mehdi Boukallel, Margarita Anastassova, Mehdi Ammi

**Affiliations:** 1 CLI Department, University of Paris 8, 93200 Saint-Denis, France; mehdi.ammi@univ-paris8.fr; 2 Laboratoire Analyse, Géométrie et Applications, University of Sorbonne Paris Nord, 93430 Villetaneuse, France; 3 Fondation Hopale, 62608 Berck, France; stephane.bouilland@fondation-hopale.org; 4 Laboratory for Integration of Systems and Technology, CEA, 91120 Palaiseau, France; mehdi.boukallel@cea.fr (M.B.); margarita.anastassova@cea.fr (M.A.)

**Keywords:** fall risk detection, wearables, data-driven deep learning, interpretable artificial intelligence

## Abstract

Injury, hospitalization, and even death are common consequences of falling for elderly people. Therefore, early and robust identification of people at risk of recurrent falling is crucial from a preventive point of view. This study aims to evaluate the effectiveness of an interpretable semi-supervised approach in identifying individuals at risk of falls by using the data provided by ankle-mounted IMU sensors. Our method benefits from the cause–effect link between a fall event and balance ability to pinpoint the moments with the highest fall probability. This framework also has the advantage of training on unlabeled data, and one can exploit its interpretation capacities to detect the target while only using patient metadata, especially those in relation to balance characteristics. This study shows that a visual-based self-attention model is able to infer the relationship between a fall event and loss of balance by attributing high values of weight to moments where the vertical acceleration component of the IMU sensors exceeds 5 m/s² during an especially short period. This semi-supervised approach uses interpretable features to highlight the moments of the recording that may explain the score of balance, thus revealing the moments with the highest risk of falling. Our model allows for the detection of 71% of the possible falling risk events in a window of 1 s (500 ms before and after the target) when compared with threshold-based approaches. This type of framework plays a paramount role in reducing the costs of annotation in the case of fall prevention when using wearable devices. Overall, this adaptive tool can provide valuable data to healthcare professionals, and it can assist them in enhancing fall prevention efforts on a larger scale with lower costs.

## 1. Introduction

Fall event prediction is a task that aims to determine how likely it is that a person might experience a fall before a fall takes place. A variety of factors can influence fall occurrence [1,2], including balance and gait disorders, cognitive impairment, visual impairment, muscle weakness, and medication use [3]. In order to assist healthcare professionals in identifying individuals who are at risk of falling, automated fall detection tools have been developed to meet this need. These tools typically involve a combination of medical history, physical examination, and an assessment of functional abilities [4]. One of these fall event assessment tools is the Timed Up and Go (TUG) test [5]. The TUG test measures the time it takes an individual to perform a series of usual tasks that require balance and gait stability, and these tasks are to stand up from a chair, walk a short distance, turn around, walk back to the chair, and sit down again. Another tool, called the Berg balance scale [6], is used to assess an individual’s ability to maintain their balance during various exercises. These tools can help identify individuals who may be at increased risk of falling.

However, some of these tools are limited by their broad-based assessment approach, which overlooks brief, fast, unstable, and irregular conduct; in addition, their accuracy in predicting fall events is not always high [7,8]. These tests may also be insufficient in terms of the number of factors that needs to be taken into account to detect this type of complex event. Furthermore, balance tests may lack the sensitivity and specificity needed to accurately identify individuals at high risk of falling. False positives and false negatives can occur, thereby leading to either unnecessary interventions for some or failure to identify fall events in others. In terms of monitoring, these tests are easily outdated, since they provide a snapshot of an individual’s balance abilities at a specific point in time. Therefore, the continuous monitoring of balance and fall risk over time may prove difficult under these conditions.

With the recent advances in artificial intelligence (AI), machine learning and deep learning models play a role in the use of novel methods for conducting fall event detection on a variety of types of data [9]. For instance, AI models were used with the data provided by wearable sensors [10], such as Inertial Measurement Units (IMUs) and instrumented insoles. These models can be trained to predict an individual’s risk of falling based on their gait patterns, and these are combined with the data on medication use, environmental hazards, and other such risk factors to provide a more accurate prediction of fall events. More specifically, wearable sensors can collect movement pattern-related data [11], and they can then detect, thanks to machine learning algorithms, the subtle changes that may indicate increased risk of falling. This can allow healthcare professionals to set up a personalized preventive rehabilitation protocol, thus preventing injury and improving the quality of life for adults and older adults.

In healthcare, there is a growing need for explainable AI (XAI). Indeed, explaining the predictions made by AI algorithms is mandatory for healthcare professionals and patients in order to check medical hypotheses and conduct assessments based on AI-powered clinical decision support systems [12]. This is particularly important in fall event prediction, where healthcare professionals need to understand the factors that contribute to an individual’s risk of falling in order to develop effective prevention strategies [13].

This study is motivated by numerous purposes, including prevention and personalized monitoring. As a matter of fact, fall event prediction has several important implications for both healthcare providers and patients. Indeed, it helps healthcare providers identify patients who are at increased risk of falling. Once this is identified, appropriate preventive measures can be implemented. This may include education on fall prevention strategies, environmental modifications, and lifestyle changes. Through this project, we also aim to leverage the progress of AI to enhance the rehabilitation process, thus guiding rehabilitation efforts by helping therapists and clinicians target specific areas of concern and design rehabilitation programs tailored to patient needs. On another note, falls can sometimes be early indicators of underlying medical conditions, such as neurological disorders or cardiovascular problems. Fall event detection based on motion data can prompt further evaluation and diagnosis [14], potentially leading to earlier treatment and improved outcomes. These measures are the building blocks of this project, and they are used to conceive a proactive and patient-centered approach to assess the risk of falling and minimize their associated consequences.

In this study, we present a data-driven approach to detect moments with the highest probability of falling by using unlabeled ankle-mounted-IMU data. Our model was trained to approach Berg balance scores, which are given metadata for all patients, in order to detect possible fall events. Since this approach relies mainly on data, we adopted the non-contrastive learning approach to allow the model to pre-train a data encoder that extracts the most informative patterns. Accordingly, the contributions of this article are summarized in the following points:

- Performing rare event detection with an interpretable deep learning architecture;
- Using a non-contrastive learning approach to improve the model’s representation of data;
- Moments with high risk of falling are highlighted by the intermediate and interpretable layers of the model instead of the output layer.

The current paper is organized as follows: Section 2 presents the related works and use cases that use different approaches to identify possible fall events, and examples that tackle the interpretability aspect. Section 3 details the configuration of the project, as well as the approach that we used for this project. In Section 4, we analyze the results of our approach and highlight the interpretability aspect of this model. Then, Section 5 draws the conclusion about the use of interpretable models in detecting fall events indirectly by using a balance score. Finally, we discuss the importance of these contributions in terms of interpretability, data-based methods, and healthcare.

## 2. Background

Identifying individuals who are at risk of falling and implementing appropriate interventions to reduce fall events can help prevent falls and their associated injuries. Risk factors [1] are to be taken into account when treating this issue. Therefore, many risk assessment tools have been developed and continuously updated for this matter [2]. Fall risk assessment is increasingly needed for older adults, as it helps in identifying the risks that endanger them. This is because advanced age is a significant risk factor for falls due to age-related changes in strength, balance, and mobility [15]. Moreover, reduced muscle strength can impair balance and increase the likelihood of falls [16]. Balance and gait problems are also major causes of falls among older adults [17]. Furthermore, medication, chronic health conditions, and visual impairment may increase the risk of falling [18,19,20]. One other aspect related to fall occurrence is cognitive impairment. In fact, environment perception can affect judgment due to cognitive decline or dementia, thus increasing the probability of falling [21].

The automation and the reliability of such tools are important factors for health practitioners. By using these frameworks as decision support tools [22], practitioners can develop targeted interventions to prevent fall scenarios. Regular threshold-based algorithms have been designed in order to extract the specific values of the used input signals, and usually, these values are computed based on the streaming signals provided by IMU sensors. Both accelerometers and gyroscopes can be used for this calculus [23]. In other works, magnetometers, quaternions, and G-force sensors have been used for the same purpose [24,25,26]. These methods have certain limitations in terms of generalizability, performance, and the number of data required for processing [27]. With the growing importance of big data in contemporary experiments, new methods have taken the lead in this type of use cases.

Recently, machine learning techniques have shown promise in healthcare, particularly in the field of gait analysis. Machine learning algorithms can analyze large numbers of data to identify patterns and relationships that may be difficult for humans to detect [28,29]. Common machine learning algorithms are particularly efficient in the detection of fall occurrences. For example, the decision tree model, known for its simplicity and easy interpretation, was able to achieve good scores in the prediction of fall risk with high probability [30]. Furthermore, the Support Vector Machine (SVM) model was able to increase the accuracy of its predictions and could also generalize more effectively for instances of unseen data [31]. Probabilistic models such as the Hidden Markov Model (HMM) are also especially efficient due to their high accuracy in predicting human falls with accelerometer data [32].

In general, many technologies have been deployed in the service of healthcare, especially with respect to gait analysis. For instance, computer vision algorithms are used to analyze video data to assess an individual’s gait, posture, and movements. These architectures can identify deviations from normal motion patterns that might indicate fall risk [33]. In the context of healthcare, data such as a patient’s history, medication details, demographics, and physical assessments are usually recorded and stored. Hence, they can be combined and processed using machine learning models as relevant features for fall assessment [34]. Additionally, deep learning models can fuse data from multiple sources, such as wearable sensors, video, and environmental sensors, to provide a comprehensive fall risk assessment [35].

More specifically, in the case of wearable sensors, deep learning architectures have also been used to solve the task of fall detection or assessment. In fact, Long Short-Term Memory (LSTM), Convolutional Neural Network (CNN), and hybrid ConvLSTM are great assets to extract temporal as well as spatial features from data in order to predict falls for older adults [36]. These types of models are fairly known for their performance in different tasks that include time series. Therefore, they are convenient when processing the data obtained from wearable sensors. Yet, deep learning models show clear limitations when it comes to interpretability due to the complex calculus that they introduce and the depth of their layers [37]. As a result, explainable AI was introduced to address this issue [13]. Attention mechanism models, also known as self-attention (SA) models, utilize an architecture that creates similarity links inside an input sequence in order to assign importance weights to its components [38]. This method has rapidly spread in different domains thanks to its interpretability, especially in healthcare, which is a field with high demand for explanatory conclusions obtained by means of model predictions. Having said this, this type of method has not yet been properly exploited for assessing fall event probabilities.

In practice, two approaches have been proposed to determine the likelihood of experiencing a fall based on the protocol setting. The first one aims to assess if the person should be considered at risk of falling or not thanks to a classification process. On the other hand, other works have proposed time-wise fall event detection that can be applied in real time or at the end of the event [39]. In the first case, studies that used the accelerometer data provided by multiple sensors mounted on different parts of the body (accompanied with pressure insoles in certain cases) showed that accuracy in fall screening ranges from 57% to 90% [40,41]. Other methods, comparable to ours, use deep learning algorithms in order to identify the samples with the highest risk of falling. In such cases, results of around 73% accuracy were achieved [42,43].

Despite the quality and novelty of the utilized approaches that aim to assess the probability of falling among older adults, certain limitations still arise. This topic is often limited by the generalization capacities of AI models, as they usually struggle to maintain a robust prediction level across diverse populations [44]. In addition, the “black-box” nature of certain AI models can make it challenging to understand the reasoning behind fall event predictions, which may be critical for healthcare providers. Finally, AI models may misinterpret non-fall-related activities or environmental factors as falls, thus leading to false alarms. Through this study, we aim to mitigate these types of limitations by building an agnostic model that is generalizable to all participants. Furthermore, we also aim to exploit the interpretable aspect of self-attention architectures to avoid instances where there is no explanation due to lack of results and to—finally—benefit from the model’s interpretability by obtaining a probability for the occurrence of a fall event (instead of a binary classification) in order to adjust a threshold of confidence that could help avoid false fall alarms in falling events.

## 3. Method

In this section, we present our framework, which uses IMU sensor data to predict possible fall events. First, we analyze the data that were collected and used for this project. Then, we explore the deep learning architecture, as well as the training pipeline. Finally, we give details about the applications of this fall event assessment tool.

### 3.1. Experimental Configuration

**Figure 1:**
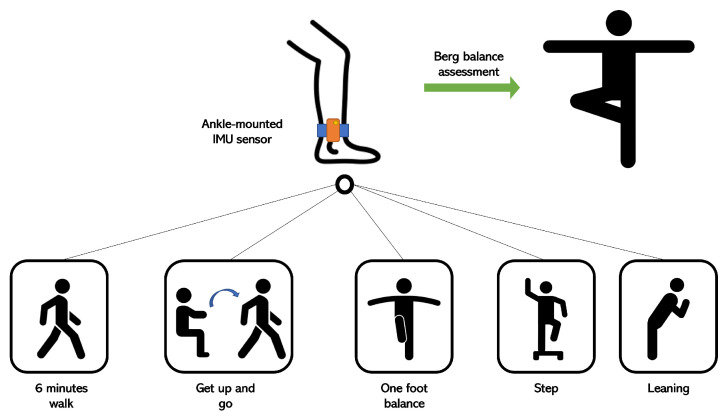
Activity descriptions.

The adopted setup for these experiments was based on accelerometer and gyroscope data. In order to acquire gait-related data, IMU sensors were mounted on the ankle of each foot. The reason behind this choice was to use the smallest number of IMU sensors as possible in places with the best relevance for gait analysis, such as the ankles. These sensors have a sampling rate of 40 Hz and a full scale of ±160 m/s² for the accelerometer component (e.g., ±2000 deg/s for the gyroscope component). In our case, the observed ranges were ±40 m/s² (accelerometer) and ±260 deg/s (gyroscope). Each patient performed a series of standardized exercises, including Get Up and Go, One Foot Balance, 6-Minute Walk, etc. Figure 1 shows the overall configuration for acquiring the data based on the sequence of the proposed exercises. The study population involved patients with a variety of paretic severities, and they were grouped into 5 categories of walking difficulty. For this project, the people who were recovering best were the most represented.

### 3.2. Data Analysis

**Figure 2:**
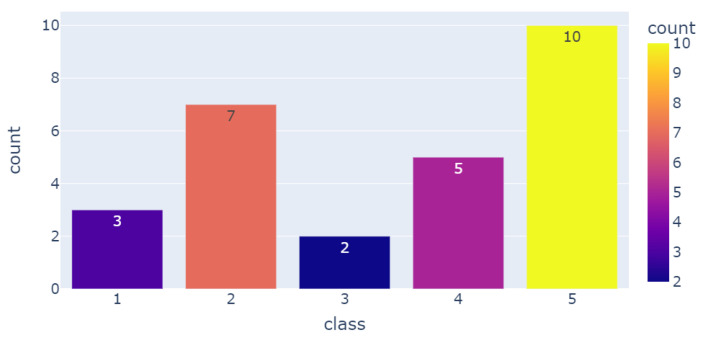
Number of patients per group of gait quality.

This study included 27 patients from Fondation Hopale in Berck-sur-Mer, France. The patients were stroke survivors undergoing rehabilitation. This dataset included patients with different motor disorders, especially hemiparesis. The sequelae of this pathology differ from one person to another, and a score of the quality of their gaits was given based on medical expertise and manual annotation: patients with severe gait disorders were given a score of 1, whereas healthy participants with a normal gait were given a score of 5. Figure 2 demonstrates the distribution of this population according to their given scores, which were based on a gait evaluation.

**Figure 3:**
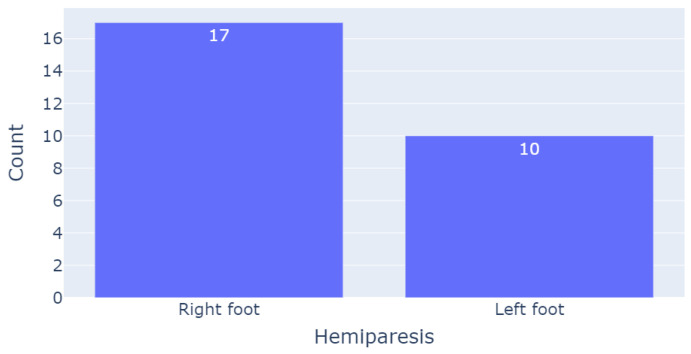
Number of patients based on the side affected by hemiparesis.

This population included participants aged from 35 years old to 84 years old, with a median age of 64 years old. There were 10 females and 16 males. The patients were being rehabilitated from 1 up to 160 months after stroke occurrence, thus making them quite varied in terms of their response to the rehabilitation process. Among the men, the mean height (in cm) and weight (in kg) were 177.7 ± 6.9 and 82.7 ± 13.1, respectively. On the other hand, the mean height and weight among women were 162.9 ± 6.7 and 66.8 ± 17.9, respectively. Regarding the medical condition of these participants, Figure 3 illustrates the number of people who were affected in their right/left foot.

Finally, some of the participants used different tools as means through which to support themselves while walking. Indeed, a support tool that had been advised based on their respective conditions was used to assist them during their rehabilitation sessions, as well as in their daily lives. Table 1 gives a detailed count of people using different types of support tools based on their paretic limb.

### 3.3. Data Preprocessing

A certain number of data are mandatory for an AI model when using the streams of data provided by IMUs for rare event detection tasks for it to be able to learn from different examples. Therefore, we clipped the recordings of the different exercises into short segments of 10 s with an overlapping sequence of 1.25 s. The duration of the recordings of each exercise is given by its mean and standard deviation in Table 2. In fact, a 10 s window of data can contain, on average, data corresponding to 6 steps, which are rich in a variety of types of behavior, repetition, and gait features. Overall, this technique helped us to acquire a consistent number of data, as well as to obtain a perfectly sized input for our model.

With respect to data representation, we propose a 3D data format in order to exploit the spatial correlation between the different axial components of the accelerometer signals and the gyroscope signals. Therefore, we used 6 different channels for the x-, y-, and z-axes for both acceleration and gyroscope data. For each channel, we reformatted the signal into a 20 × 20-sized matrix by clipping it into 20 equally sized sequences of length 20. Then, we concatenated them as rows of a new 2D matrix. We next completed the same process for each signal component (Acc_x, Acc_y, Acc_z, Gyr_x, Gyr_y, and Gyr_z). Then, we superimposed the resulting 6 matrices into a single 3D matrix, where each channel corresponded to the 2D matrix of a signal component. At the end, each input observation for the model was of size 20 × 20 × 6.

### 3.4. Pre-Training Target Variable

**Figure 4:**
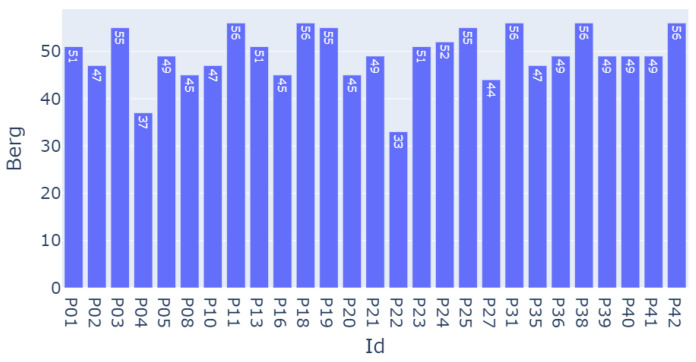
Berg assessment score for each patient.

Our model consists of a pre-training phase where the model trains to approach a Berg balance assessment score. This score was the result of an evaluation of a series of equilibrium exercises that were performed by the participants. The values of this score for the participants varied from 33 to 56, as demonstrated in Figure 4.

With respect to this, two possibilities were proposed for creating the training pipeline and defining the task type:

- Regression problem: By keeping the Berg score in its current format, the developed model could have been trained to predict the exact value of this score;
- Classification problem: Transforming the balance score into the three categories of the intervals 30–40, 41–50, and 51–60 could have allowed the model to be trained on a multi-label classification task.

The second configuration was less effective in terms of training progress, and the loss function was especially unstable. In addition, the results of the classification were not sufficient. On another note, the first approach allowed for smoother training while also closing the gap between the model’s predictions and the real values of the Berg assessment score. Consequently, the following sections involve the findings from the regression task alone.

### 3.5. Self-Attention Architecture

**Figure 5:**
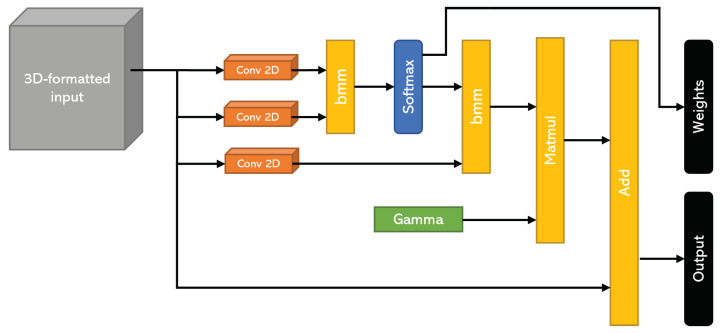
The spatial self-attention architecture.

As detailed previously, this model uses a balance score for training and conceiving a compact and informative representation of data along with corresponding importance weights. In order to extract these weights, we used a spatial self-attention mechanism, which was implemented with convolutional layers. This type of architecture can highlight the correlations among the parts of the signal itself, as well as with other components. In our case, this was the most apt configuration, since we sought to pinpoint the instants of the signal that explained the most of the Berg score. In this way, we hoped to detect the instants with the highest probability of predicting a fall. Figure 5 illustrates the self-attention architecture that was used in the pre-training phase. This architecture consists of three similar convolution layers, each of which computes key, query, and value. By computing the softmax of the dot product between the key and query, we obtained the similarity patterns between them, which were used as descriptive attention scores. On the other hand, we used the value representation to compute the weighted values of the input, which were multiplied by the attention scores. We used the gamma parameter in order to allow the model to give more or less importance to the self-attention module in creating the feature maps for the overall network. This meant that for high values of gamma, the outputs were more influenced by the self-attention module.

### 3.6. Non-Contrastive Learning Approach

**Figure 6:**
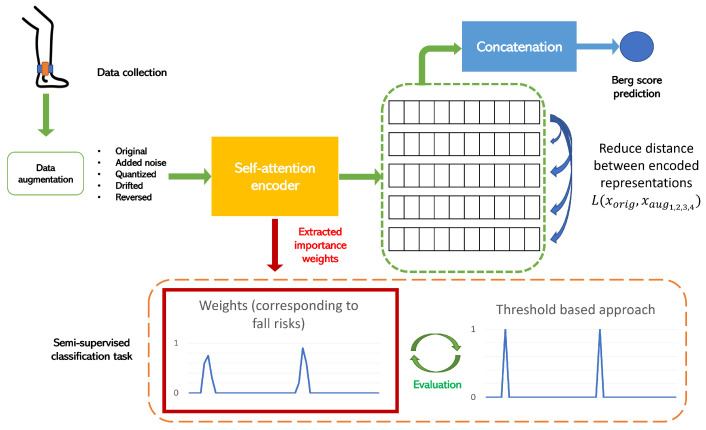
The full pipeline of the non-contrastive learning approach.

The self-attention mechanism was developed in order to be used for a non-contrastive learning approach. In Figure 6, we illustrate the steps of this approach. These consisted in creating different versions of the input data by using the following types of data augmentation:

- White noise: We added randomized white noise along the different time series, and this noise was simply a random Gaussian signal with a mean that was computed for each signal;
- Random quantization: This approach maps the values of the continuous signal into smaller sets of discrete values with a chosen step;
- Drift: In this case, the algorithm randomly and smoothly drifts the values of a time series from its original values;
- Reverse: This approach simply involves reversing the time series and rendering it backwards.

Therefore, we obtained the original time series and 4 more variants of it thanks to the augmentation techniques. After that, we extracted the data representation that was given by the self-attention encoder for the original input, as well as the four augmented versions. Then, we trained the model to learn the similarities among the rendered representations. Simultaneously, we concatenated the inputs’ representations and provided them to a regression layer that predicted the Berg score assessment. In this way, we allowed the model to undergo a two-fold effect when learning data representation, which is meaningful in terms of equilibrium, and extracted the weights of correlation between the input signals and the loss of equilibrium, which is a direct cause of falling for older adults.

This method allows us to detect rare events, which are fall risk moments in our case, with unlabeled data. Furthermore, we only used the Berg score labels in the pre-training phase. By extracting the important sequences from the signal thanks to the self-attention module, we aimed to extract the moments that correlated with the Berg score, such that we could compare them with the phases with the highest risk of falling without any dedicated supervised training for this task. Then, we compared these results with a basic threshold-based approach for detecting the risk of falling based on accelerometer data. The combination of the non-contrastive learning approach and the self-attention mechanism is a novel way with which to solve classification problems in the detection of rare events and to avoid the problems of huge imbalances in data. Moreover, by means of interpretable correlations, this model is able to create a link between the Berg score and the loss-of-equilibrium moments leading to fall possibilities.

### 3.7. Training Configuration

Considering the fact that we trained the model on two tasks simultaneously, we used two different losses that were adapted for each case. For the score prediction, we used the simple mean absolute error, which is defined by the following equation: $l_{n}=\left|x_{n}-y_{n}\right|$. On the other hand, we used contrastive loss, given by y×d+(1−y)×max(margin−d,0), where *d* is the Euclidean distance between the two samples, demonstrated as follows: $d=\left|\right|x_{1}-x_{2}\left|\right|$. Since our final task was the detection of potential falling moments without the use of annotated data, we had to visualize the weight maps for the samples of certain different configurations in order to have additional material to evaluate the performance of our model. The training phase included different experiments in order to find the most efficient hyperparameters for our models. Therefore, we used a grid search process to compare the performance of each configuration, as well as to extract the most fitting hyperparameters for our use case. On one hand, we varied the values of the learning rate, batch size, and number of epochs for the training process. On the other hand, we chose the kernel sizes in the CNN-based self-attention module to be evaluated. The search space for each of the different hyperparameters is given in Table 3.

The series of experiments were launched on a DELL laptop with the following characteristics: 11th Gen Intel(R) Core(TM) i9 (2.60 Hz), 32Go on RAM, and NVIDIA RTX A4000 graphics card. We used the “Pytorch” library for the development of deep learning architectures, “sickit-learn” for model evaluation, and the “tsaug” package to generate the augmented data in the pre-training phase. As for the training session duration, depending on the combination of hyperparameters selected by the grid search algorithm, it varied between 17 min, 5 s and 3 h, 41 min.

### 3.8. Partitioning and Evaluation

To guarantee a diversified distribution of samples for both training and evaluation, we split the data per patient and per activity in an 80–20% ratio and then added them to the test and training sets, respectively. Accordingly, the overall size of the partitions was also split in an 80–20% ratio. Usually, 80% of the data are used for the training process. To demonstrate the potential of our method, we used only 20% of the data for the non-contrastive learning training phase; we then evaluated the model on the remaining 80%. The evaluation of the model required a ground-truth reference for comparison. Thus, we used one of the baselines of the fall event assessment tools to train the threshold-based approaches. Indeed, we compared our results with the predictions provided by an algorithm that extracts moments in which a sudden change in acceleration is detected in the vertical direction, which, in our case, was where the y-axis signal was concerned. The threshold of the values of acceleration was fixed at 5 m/s², and this value was approximated at 0.5 g (where g is the gravitational constant and its value is 9.81 m/s²). Many works have used this value as a minimum threshold for the beginning of the free-fall phase; ref. [45] used 0.4 g in order to provide an alert during the pre-fall zone so as to avoid injury. Other approaches aim to find sequences where an abrupt change in acceleration is observed [46] (i.e., a variation from 5 m/s² to 15 m/s² in a short period of time, which—more specifically—is 300 ms).

## 4. Results

**Figure 7:**
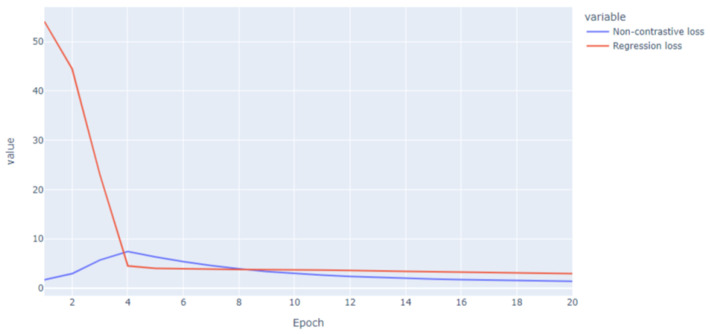
Loss functions for the non-contrastive learning and regression tasks.

We chose our best model based on its loss evolution, its capacity to approach the Berg score, and the extracted weights for the detection of fall events. First, the loss functions (shown in Figure 7) were both stable and decreased smoothly through the epochs. In other cases, they had known fluctuations and unstable evolution in time. On rare occasions, the loss curves, especially the non-contrastive loss, diverged, and the model was not able to extract the pertinent weights. For this chosen model, the fixed parameters are detailed in Table 4.

**Figure 8:**
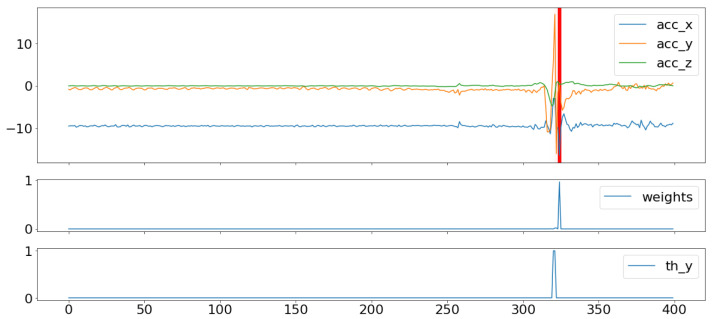
Weight projection on the IMU signals for one risk event. (The red line represents the projection of high values of weights).

**Figure 9:**
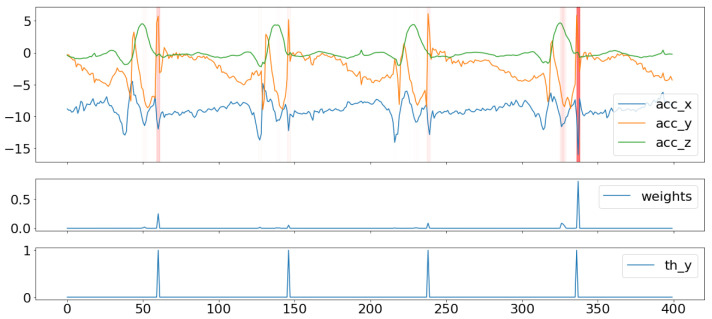
Weight projection on the IMU signals for a multitude of risk events with different importance weights. (The red lines represent the projection of high values of weights).

We used the second output of the model, which is the weight of each timestamp of the signal in explaining the Berg score in each trial. Figure 8 shows an example of the projection of these weights on the signal, and we superimposed this figure with the plot of the weights, as well as with the reference labels that indicate the instants where the axial components of the acceleration signal exceeded the value of 5 m/s². In other cases, the model could also track a multitude of risk possibilities, although it was also able to accord more importance to potential falls with the highest risk, as demonstrated in Figure 9.

**Figure 10:**
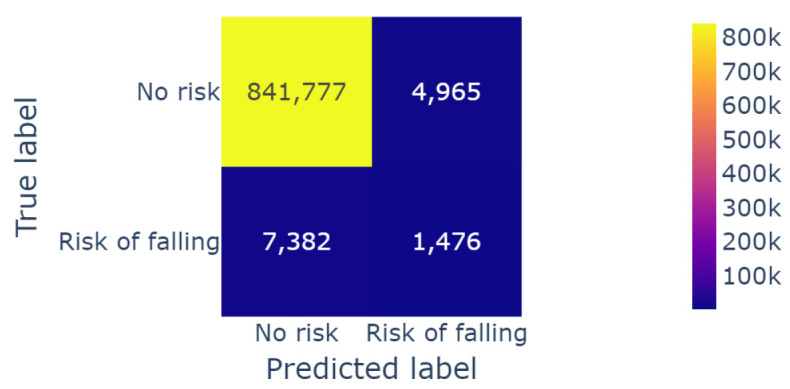
Confusion matrix for the fall risk instants.

The model was able to perfectly predict 1476 cases of possible falls out of the 8858 cases that were computed by the threshold approach. The overall accuracy of the model reached 98.9% due to the imbalanced classes of the data. The confusion matrix (shown in Figure 10) listed the correctly labeled risk moments, as well as the mislabeled data. Therefore, we focused our analysis on the “fall risk” class.

**Figure 11:**
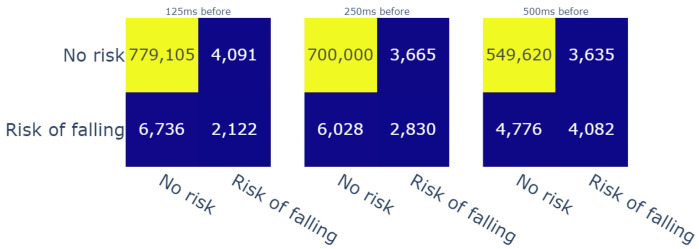
Confusion matrices for the different durations of windows of time (before reference).

**Figure 12:**
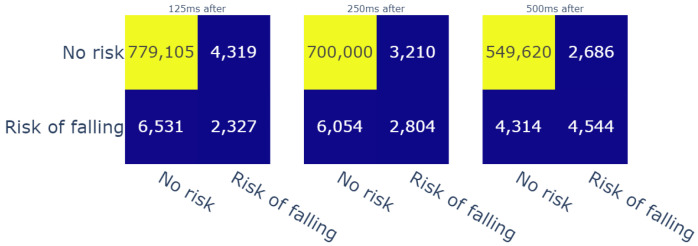
Confusion matrices for the different durations of windows of time (after reference).

**Figure 13:**
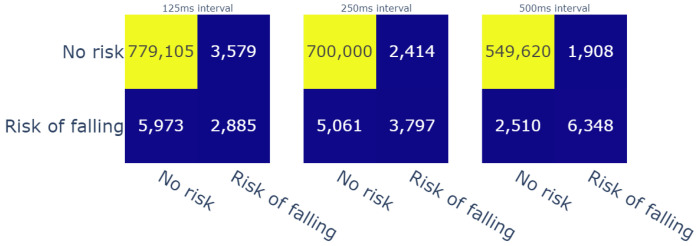
Confusion matrices for the different durations of window of time (confidence interval).

Due to the fact that our model detects specific instants of fall risk on a temporal basis, our prediction might predict fall moments before or after the true labeled risk instant. In cases where the model’s prediction of risk is completed before the ground-truth prediction, the prediction is beneficial to the assessment tool from a preventive point of view. Moreover, if the model predicts the possibility of falling after its occurrence, certain methods suggest spreading the prediction on a small window, which we usually call a confidence interval. For this reason, we evaluated the True Positives (TPs), True Negatives (TNs), False Positives (FPs), and False Negatives (FNs) at a window level while comparing the different sizes of evaluation windows. We selected three modes of evaluation, and the following are the possible scenarios:

- Predicted value inside a window of time before the reference label (Figure 11);
- Predicted value inside a window of time after the reference label (Figure 12);
- A confidence interval, in which the reference label is in the middle of the window of time (Figure 13).

For therapists, detecting risk of falling in advance is particularly important from a preventive standpoint. Furthermore, intelligent fall prevention systems usually aim to launch alerts during the transition phase, i.e., between the “Activity of daily living” (ADL) phase and the free-fall zone. This transition phase can last an especially short time span, which varies from 150 ms [47] to 300–500 ms [48] (depending on mobility factors for each patient). Given these durations, we evaluated the predictions of our model with three temporal window sizes, i.e., 125 ms, 250 ms, and 500 ms, before the computed labels. The achieved results showed that the model detected up to 4082 fall risk moments (c.f. Figure 11). On another note, we used the same temporal windows to evaluate the risk events that were detected moments after the reference risk events. In this case, for the same temporal windows, the model detected up to 4544 risk moments (c.f. Figure 12). In addition, we chose to create a confidence interval that included the results before and after the references with respect to the same evaluation window sizes in order to have an overall idea about the performance of the model. In that regard, the model was able to detect 6348 out of 8858 fall (c.f. Figure 13) risk events, which represent 71.6% of the cases. Thanks to this evaluation process, we gained more than 55% in the model’s recall score. In addition, the precision of the model reached 77%. These scores were given for weights that were higher that 0.1 compared with recall of 9% and precision of 98%; however, this applied only if the weights higher than 0.5 were taken into account. This means, in that case, that the model detected fewer fall risk events (around 830 occurrences). On the other hand, it did produce fewer false negatives (only 11). Therefore, finding the best threshold was also mandatory for this study, and fixing it at 0.1 helped to obtain an acceptable ratio of correctness in the model’s predictions.

## 5. Conclusions and Perspectives

In this paper, we propose a non-contrastive learning approach to predict, using the Berg balance assessment score, fall risk for both adults and older adults who are stroke survivors. Its advantages can be summarized in the following points:

- It can be trained on a small number of unlabeled data;
- It uses interpretable architectures on metadata to extract meaningful features;
- The model is used to exploit the cause–effect link between the balance score and the target variable.

The results obtained were particularly interesting in terms of the quality of detection. In fact, the model reached a recall score of 71% and a precision score of 77% in cases with a 1 s confidence interval (specifically 500 ms before and after the reference prediction). It is important to keep in mind that the model was not trained to detect these risk events in a direct and supervised way. Instead, it was trained to provide approximations of the Berg score and to use the interpretability assets of the model in order to find the link between the balance score and the risk of falling. This was achieved by extracting the importance weight of each timestamp, and it appeared that the model pinpointed instants with extreme values of acceleration and the sudden evolutions within them. Therefore, the model achieved decent scores, especially with respect to the fact that the model itself extracts the relation between the balance score and the values of acceleration in order to determine fall risk moments without the use of any labeled data for this purpose. Moreover, the threshold detection approach is well grounded and is shown to achieve good scores, yet it may show certain weaknesses in terms of generalizability and adaption to profiles, i.e., the reference labels are not especially reliable. In another vein, this study still has many directions in which there could be possible improvements. The proposed CNN-based self-attention model was only compared with the threshold approach. Other explainable architectures could be tested and adapted for this use case, and the model of this study could be compared with more reliable detection approaches or annotated data. Furthermore, the non-contrastive learning pipeline needs to be trained on large datasets in order to extract different types of patterns and information. Moreover, the contrastive self-supervised learning method could be tested for this problem, specifically in terms of allowing the model to distinguish between normal patterns of gait and abnormal sequences that may lead to falling. From a practical point of view, we observed that the model had a tendency to predict fall risk in the interval that preceded the reference labeling. Thus, our approach could serve as a prevention tool for fall risk assessments. In terms of utility, this project aims to conceive an interpretable tool for decision making to assist health practitioners in their daily tasks, especially for gait analysis and fall risk management in the elderly. Its varied purposes could evolve around profiling patients and suggesting adapted rehabilitation indicators, including the use of gait phases to improve the prevention of patient falls, or the use of a type of walking support and the adequate moment for changing it. Furthermore, this tool is bound to be used for monitoring patients in different configurations aside from clinical rehabilitation sessions. In order to meet these needs, we will work on building a more robust model that allows one to also have an interpretable output based on the inputs. By adding more metadata and information from patient records, the model could gain more efficiency and trustworthiness to reassure tool users. In addition, it could also help to enhance its interpretable outputs with indicators that are more relevant to the medical staff.

## Figures and Tables

**Table 1:** Number of walking support users.

Type of Support	Paretic Side	
	**Left Foot**	**Right Foot**
Cane	7	2
Tripod cane	1	1
Molded lifter	1	3
Rubber band lifter	0	2

**Table 2:** Mean duration of the recording of each exercise.

Activity	Duration	
	**Mean**	**Std. Dev.**
6-Minute Walk	6 min 31 s	13 s
Get Up and Go	50 s	29 s
One-Foot Balance	53 s	31 s
Step	46 s	27 s
Leaning	1 min 11 s	6 s

**Table 3:** Search space for the model’s hyperparameters.

Hyperparameters	Range of Search Space
Batch size	[1, 4, 8, 16, 32]
Learning rate	[1 × 10^−2^, 1 × 10^−3^, 1 × 10^−4^, 1 × 10^−5^]
Number of epochs	[5, 10, 15, 20, 30, 40, 50]
Self-attention kernel size	[1, 2, 4, 6, 8]

**Table 4:** Optimized hyperparameters.

Hyperparameter	Batch Size	Learning Rate	Epochs	Kernel Size
Optimized value	4	1 × 10^−3^	20	1

## Data Availability

Data available on request.
